# Disease-related mutations among Caribbean Hispanics with familial dementia

**DOI:** 10.1002/mgg3.85

**Published:** 2014-06-04

**Authors:** Joseph H Lee, Amanda Kahn, Rong Cheng, Christiane Reitz, Badri Vardarajan, Rafael Lantigua, Martin Medrano, Ivonne Z Jiménez-Velázquez, Jennifer Williamson, Peter Nagy, Richard Mayeux

**Affiliations:** 1 The Taub Institute for Research on Alzheimer's Disease and the Aging Brain, Columbia UniversityNew York, New York; 2 The Gertrude H. Sergievsky Center, Columbia UniversityNew York, New York; 3 The Department of Epidemiology, School of Public Health, Columbia UniversityNew York, New York; 4 The Department of Neurology, Columbia UniversityNew York, New York; 5 The Department of Medicine, College of Physicians and Surgeons, Columbia UniversityNew York, New York; 6 School of Medicine, Pontificia Universidad Catolica Madre y MaestraSantiago, Dominican Republic; 7 Department of Internal Medicine, University of Puerto Rico School of MedicineSan Juan, Puerto Rico; 8 The Department of Pathology, Columbia UniversityNew York, New York; 9 The Department of Psychiatry, Columbia UniversityNew York, New York

**Keywords:** Alzheimer's disease, Caribbean Hispanics, familial dementia, mutations, next-generation sequencing

## Abstract

Pathogenic mutations in the three known genes – the amyloid precursor protein (*APP*), presenilin 1 (*PSEN1*), presenilin 2 (*PSEN2*) – are known to cause familial Alzheimer's disease (AD) and tend to be associated with early-onset AD. However, the frequency and risk associated with these mutations vary widely. In addition, mutations in the frontotemporal lobar degeneration (FTLD) genes – the microtubule-associated protein tau (*MAPT*), granulin (*GRN*) – have also been found to be associated with clinical AD. Here, we conducted targeted resequencing of the exons in genes encoding *APP*, *PSEN1*, *PSEN2*, *GRN*, and *MAPT* in 183 individuals from families with four or more affected relatives, presumed to be AD, and living in the Dominican Republic and Puerto Rico. We then performed linkage and family-based association analyses in carrier families, and genotyped 498 similarly aged unrelated controls from the same ethnic background. Twelve potentially pathogenic mutations were found to be associated with disease in 53 individuals in the five genes. The most frequently observed mutation was the *p.Gly206Ala* variant in *PSEN1* present in 30 (57%) of those sequenced. In the combined linkage and association analyses several rare variants were associated with dementia. In Caribbean Hispanics with familial AD, potentially pathogenic variants were present in 29.2%, four were novel mutations, while eight had been previously observed. In addition, some family members carried variants in the *GRN* and *MAPT* genes which are associated with FTLD.

## Introduction

In 2001, a founder mutation, *p.Gly206Ala* (g.44636G>C) variant in presenilin 1 (*PSEN1*) (MIM#: 104311; NM_000021.3:c.617G>C) was identified in a group of families from Puerto Rico and the Dominican Republic (Athan et al. 2001). There are now approximately 70 known families with this mutation and they are all of Caribbean Hispanics ancestry. Interestingly, some families conform to a Mendelian inheritance pattern with multiple affected family members in more than one generation. Moreover, mutation carriers can have the ages of onset ranging from 40 to 73 in these families, while the family members may have age at onset as late as in their 90s.

Studies have shown that phenotypic expression associated with any nonsynonymous variants can be highly variable (Cruts et al. 2012). Among Caribbean Hispanics, the *p.Gly206Ala* variant in *PSEN1* was highly prevalent among familial Alzheimer's disease (AD) and at the same time was associated with highly variable onset age. Furthermore, mutations in the genes associated with frontotemporal lobar degeneration (FTLD), namely the microtubule-associated protein tau (*MAPT*) and progranulin (*GRN*) genes were also found to be associated to clinical AD (Wojtas et al. 2012). We were thus motivated to conduct targeted resequencing of exonic regions within five known genes (*PSEN1* [MIM#: 104311; NM_000021.3], *PSEN2* [MIM#: 600759; NG 007381; NM_000447.2], *APP* [MIM#: 104760; AC 000153; NM_000484.3], *MAPT* [MIM#: 157140; NG 007398; NM_016835.4], and *GRN* [MIM#: 138945; NG 007886; NM_002087.2]) related to familial dementias in Caribbean Hispanic families.

All families were recruited as part of a study of familial AD, and included individuals with both early (below age 65 years) and late-onset (above age 65 years) disease. To determine whether variants in any of the five known genes contributed to development of familial AD, we sequenced one case from each family, and then genotyped nonsynonymous variants in all sampled family members as well as a group of unrelated 498 controls of Caribbean Hispanic ancestry. This study provides information about clinical relevance of rare variants observed in genes associated with familial dementias found in a unique set of Caribbean Hispanics.

## Methods

### Subjects

We selected 183 patients with dementia from families of Caribbean Hispanic ancestry with four or more individuals affected with dementia, having met criteria for probable AD (McKhann et al. 1984). Each of these families had at least one affected individual with age of onset before 65 years while other family members could have later ages at onset. We further restricted the selection to a single member from each family that had multiple affected individuals, the selection was carried out based on DNA availability and, in case of more than one affected obtainable, we choose the younger one. We sequenced all exons of five genes, specifically *PSEN1*, *PSEN2*, *APP*, *GRN*, and *MAPT*. For any putative variant (selected as potentially affecting proteins function as described below), we confirmed the variant in the person sequenced and then genotyped the variant in all family members, totaling 398 samples.

We genotyped 498 unrelated and unaffected elderly Caribbean Hispanic controls if at least one additional affected family member had the genotyped variant. This was done in order to compare the allele frequency in a healthy Caribbean Hispanic cohort.

### Next-generation sequencing

High-throughput sequencing was performed using the Roche 454 GS FLX sequencing platform (Roche 454 Life Sciences, Branford, CT). Exonic sequences from the five genes of interest were amplified using Fluidigm capture technology (http://www.fluidigm.com).

### Sequencing analysis

Data were analyzed using NextGENe software from SoftGenetics (SoftGenetics, LLC, State College, PA). Reads were filtered based on the following criteria: the median quality score was below a threshold of Q15; more than three consecutive uncalled bases, there were less than 25 called bases per read. The reads were then aligned against GenBank reference file, version GRCh37.p5 primary assembly, for the five targeted genes. The average read length was approximately 480 basepairs, and the average coverage per subject was approximately 30×. We required a minimum of 10-fold coverage and the presence of the mutation in greater than 15% of the reads or at least three reads, for a heterozygous variant to be called at any nucleotide. Mutation reporting was restricted to coding DNA regions and splice sites. Nonsynonymous variants designated by SIFT (Ng and Henikoff 2001; Kumar et al. 2009) as “damaging” were selected for further genotyping analysis.

### Genotyping

We regenotyped the sequenced cases using the Sanger method to validate genotypes obtained from the sequencing experiment. Similarly, we genotyped their family members with available DNA to establish disease segregation patterns within each family. Genotyping was conducted on the Sequenom platform, and was supplemented with the Sanger method. Variants that were likely segregating within families were genotyped in 498 unrelated controls of the same Caribbean Hispanic ancestry who underwent the same phenotype and diagnostic review.

### Statistical analysis

We performed linkage and family-based association analyses using PSEUDOMARKER (Goring and Terwilliger 2000; Hiekkalinna et al. 2011) in a dominant model, which allowed analysis of family data, unrelated subjects, or both to determine whether a variant was associated with dementia. Unlike traditional linkage analysis that computes the likelihood of recombination fraction alone or traditional allelic association analyses that often require unrelated individuals, this likelihood-based approach estimates the recombination fraction between trait and single nucleotide polymorphisms (SNPs), SNP allele frequencies, and linkage disequilibrium (LD) between trait and SNPs. As a result, it is possible to test hypotheses of linkage, LD or both. Moreover, by including unrelated controls in a family study, this approach provides more accurate allele frequencies in the general population and overcomes the problems associated with inflated allele frequencies of rare variants in highly selected families as these family members share genotypes identical by descent (Wijsman et al. 2011). Thus, we included 498 unaffected and unrelated elderly controls in the analysis because allele frequencies in multiplex families are often inflated compared with the generation population. Considering 12 variants analyzed and a significant threshold of *P* = 0.05, a Bonferroni corrected *P* value of 0.004 was considered significant.

To determine the degree of risk associated with these variants, we performed a univariate Cox proportional hazard model adjusted for family membership as an aggregate to take into account nonindependence among family members. We then performed an association model that included *APOE* (modeled as having at least one copy of APOE*4). The proc PHREG module in SAS (SAS Institute Inc., Cary, NC) was used for the analysis.

## Results

### Subjects

Probands from 183 families were sequenced (Table 1). There were 67.2% women and the mean age of onset was 56.6 years (SD = 6.94), ranging from 30 to 64 years. The mean number of years of education was 8.4 years (SD = 5.5). Thirty-four percent of the probands were from Puerto Rico, 65% from the Dominican Republic, and one was born in the United States. The APOE*4 allele frequency was 32.3%. In addition, 498 controls of similar ancestry were genotyped. There were 69.5% women and the mean age at last examination was 79.1 years (SD = 6.2) and ranged from 66 to 100 years. The age at onset for sequenced individuals ranged from 40 to 73, while that for the relatives of the sequenced individuals ranged from 44 to 98. For unrelated controls, age at last examination ranged from 42 to 100. The mean number of years of education was 7.6 years (SD = 4.4). The distribution of reported country of origin for controls was as follows: 49.2% were from Dominican Republic, 16.6% from Puerto Rico, 18.9% from Cuba, and the remainder was from other Latin American countries. The APOE*4 allele frequency was 13.0%.

**Table 1:** Demographic and clinical characteristics of sequenced participants and family members

	Sequenced		Relatives		Controls	
			
Characteristics	*n*	Mean	*n*	Mean	*n*	Mean
Number of subjects sequenced/genotyped^1^	195		318		498	
Number of families^2^	194		56			
Affection status						
Affected	183	94.0%	104	33.0%		
Unaffected	10	5.0%	197	62.0%	498	100.0%
Unknown	2	1.0%	17	5.0%		
Proportion of females	64/131	67.2%	142/176	55.30%	152/346	69.5
Age (year)						
Age at onset of affected		56.9 (SD = 7.29)		68.5 (SD = 12.64)		
Age at last examination of unaffected		66.7 (SD = 5.66)		58.8 (SD = 11.31)		79.1 (SD= 6.2)
Range of age at onset		40–73		44–98		
Education (year)		8.4 (SD = 5.45)		90 (SD =5.79 )		7.6 (SD = 4.35)
Range of education		0–20		0–22		0–20
Residency						
Puerto Rico	67	34.4%	147	46.2%	69	13.9%
Dominican Republic	127	65.1%	165	51.9%	210	42.2%
USA	1	0.5%	0	0.0%	10	2.0%
Other Hispanics^3^	0	0.0%	0	0.0%	132	26.5%
Unknown/others^4^	0	0.0%	6	1.9%	77	15.5%
APOE allele frequencies						
E4		32.1%		24.1%		13.0%
E3		64.4%		70.9%		79.2%
E2		3.6%		5.0%		7.8%

1 Three individuals identified from separate sequencing experiments.

2 One family had more than one person sequenced.

3 Self-reported Hispanics but unclear about their nationality and place of birth.

4 Incomplete information.

### Sequence analyses

Sequencing revealed a small number of variants. Twelve nonsynonymous variants were considered damaging (Ng and Henikoff 2001; Kumar et al. 2009). Variants were present in all five genes among cases, but were either absent or extremely rare among unrelated controls (Table 2).

**Table 2:** Comparison of mutation frequencies between affected versus unaffected family members versus unrelated controls

Mutation		Affected			Unaffected			Unknown			Controls		
					
Gene	Total^1^	No. of carriers	Total aff	freq	No. of carriers	Total unaff	Freq	No. of carriers	Total unk	Freq	No. of carriers	Total controls	Freq
*PSEN1*													
*p.Gly206Ala*	600	118	303	0.195	71	259	0.137	14	38	0.184	0	498	0.00000
*p.Glu318Gly*	222	9	198	0.023	4	22	0.091	0	2	0	13	498	0.01305
*PSEN2*													
***p.Ile235Phe***^2^	***196***	***1***	***183***	***0.003***	***2***	***11***	***0.091***	***0***	***2***	***0***	***0***	***498***	***0.00000***
***p.Pro301Ala***^2^	***198***	***1***	***184***	***0.003***	***2***	***12***	***0.083***	***0***	***2***	***0***	***0***	***497***	***0.00000***
***p Ala344Val***^2^	***195***	***1***	***184***	***0.003***	***1***	***9***	***0.056***	***0***	***2***	***0***	***1***	***497***	***0.00101***
*GRN*													
***p.Cys222Tyr***^2^	***193***	***1***	***183***	***0.003***	***0***	***8***	***0***	***0***	***2***	***0***	***0***	***498***	***0.00000***
*p.Val519Met*	201	4	186	0.011	2	13	0.077	0	2	0	0	498	0.00000
*MAPT*													
*p.Ser318Leu*	264	23	220	0.052	14	39	0.179	1	5	0.100	53	498	0.05522
*p.Ile468Thr*	194	1	184	0.003	1	8	0.063	0	2	0	2	497	0.00201
*APP*													
*p.Ser614Gly*	192	1	183	0.003	0	7	0	0	2	0	8	498	0.00803
*p.Ala344Val*	195	2	184	0.005	0	9	0	0	2	0	0	498	0.00000
*p.Val340Met*	198	3	186	0.008	2	10	0.100	0	2	0	0	498	0.00000

1 Total number of subjects sequenced or genotyped.

2 Variants in bold italics were not found in Alzheimer's disease and frontotemporal dementia mutation database (http://www.molgen.ua.ac.be/ADMutations).

### Presenilin 1

The *p.Gly206Ala* variant (NM_000021.3:c.617G>C) in *PSEN1* was the most frequent and had the strongest effect on the risk of dementia (Table 3). None of the controls had this mutation. This mutation showed strong evidence for both linkage as well as association (Table 3) either in the presence or absence of controls. A second mutation *p.Glu318Gly* variant (NM_000021.3:c.953A>G) in *PSEN1* was observed in three families. The allele frequency among unrelated controls was 1.3%. This variant showed no evidence for linkage, but was associated with dementia when the cases were compared with the unrelated controls, suggesting that this variant may be a risk factor.

**Table 3:** Joint linkage and association analysis using families and 498 unrelated controls

			*P*-values (with unrelated controls)	
			
Gene	Variant name	MAF in controls	Linkage^1^	LD and linkage^2^
*APP*	*p.Ala344Val*	0	0.1195	0.0005
	*p.Ser614Gly*	0.008	0.4958	0.0107
	*p.Val340Met*	0	0.5	0.0007
*PSEN1*	*p.Glu318Gly*	0.0131	0.3805	6.0E-6
	*p.Gly206Ala*	0	8.94E-07	<1.0E-24
*PSEN2*	*p.Ala344Val*	0.001	0.2717	0.003
	*p.Ile235Phe*	0	0.5	0.0005
	*p.Pro301Ala*	0	0.5	0.0017
*GRN*	*p.Cys222Tyr*	0	0.5	0.0005
	*p.Val519Met*	0	0.4915	6.8E-08
*MAPT*	*p.Ile468Thr*	0.002	0.3017	0.0054
	*p.Ser318Leu*	0.0552	0.4893	2.4E-05

LD, linkage disequilibrium; *APP*, amyloid precursor protein; *PSEN1*, presenilin 1; *PSEN2*, presenilin 2; *GRN*, granulin; MAPT, microtubule-associated protein tau; MAF, minor allele frequency.

1 *P*-values for linkage analysis.

2 *P*-value for joint linkage and LD.

### Presenilin 2

Three damaging mutations were observed, including *p.Ala344Val* (NM_000447.2:c.1130C>T), *p.Ile235Phe* (NM_000447.2:c.703A>T), and *p.Pro301Ala* (NM_000447.2:c.1000C>G). Evidence for linkage was weak; however, the associations were significant in the joint linkage and association analysis.

### Amyloid precursor protein

Three variants were observed in this gene. Two variants, *p.Ser614Gly* (NM_000484.3:c.1840A>G) and *p.Ala344Val* (NM_000484.3:c.1031C>T) in *APP* were considered tolerated and not pathogenic. The *p.Val340Met* (NM_000484.3:c.1018G>A) variant was predicted to be nontolerated according to the SIFT evaluation, and was present in three affected and two unaffected individuals and was significantly associated with AD.

### Granulin

Both *p.Val519Met* (NM_002087.2:c.1555G>A) and *p.Cys222Tyr* (NM_002087.2:c.665G>A) variants in *GRN* were significantly associated with AD compared with the unrelated controls under the three different models (*P* < 6.8E-08 and *P* < 5E-04, respectively). None of the controls carried these two variants.

### Microtubule-associated protein tau

While a variant in *MAPT*, *p.Ser318Leu* (NM_016835.4:c.953C>T), was also frequent (5.5%) among controls as shown in Table 3, the allelic association with AD was significant; however, the other rare variant, *p.Ile468Thr* (NM_016835.4:c.1403T>C), was not.

### Cox proportional hazard model

**Figure 1:**
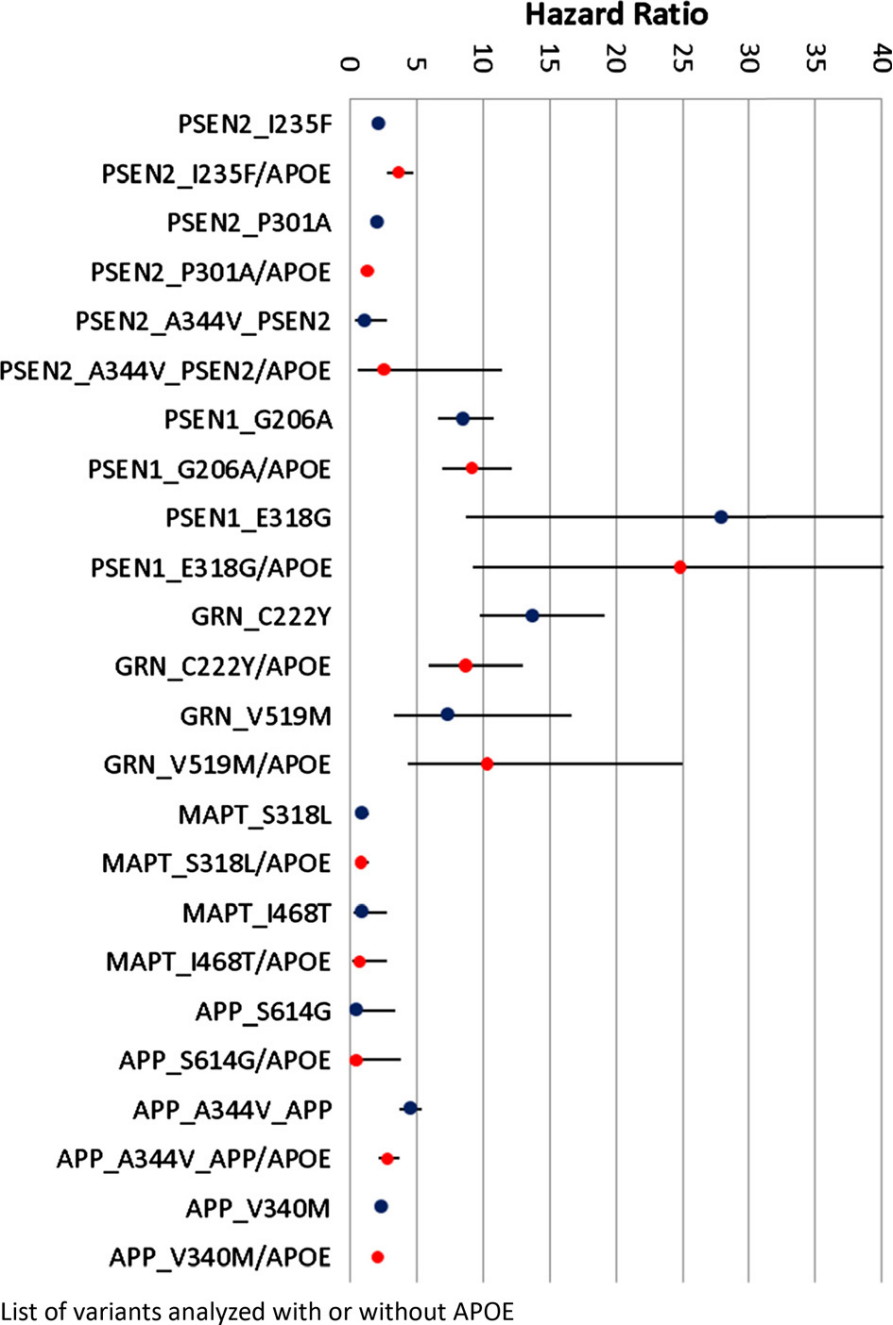
Univariate and multivariate hazard ratio of Alzheimer's disease among carriers and noncarriers using affected and unaffected individuals. Blue dots represent hazard ratios from a univariate model and red dots represent hazard ratios from a multivariate model (SNP + sex + APOE). On *y*-axis, gene and mutation are presented; horizontal bars represent 95% confidence interval. For *p.Glu318Gly* variant in *PSEN1*, 95% confidence interval was truncated at 40.

To determine the effect size of each variant in this selected set of familial AD, we performed two Cox proportional hazard models. Compared with unrelated controls, four variants under the univariate analysis had hazard ratios exceeding 5.0: the *p.Gly206Ala* variant in *PSEN1* (HR = 8.5, 6.6–10.9); the *p.Glu318Gly* variant in *PSEN1* (HR = 27.9, 9.5–82.0), the *p.Cys222Tyr* variant in *GRN* (HR = 14.4; 10.8–19.2), and the *p.V519* variant in *GRN* (HR = 7.2; 3.2–16.1). While statistically significant at *P*-value of 0.05, the hazard ratio for the *p.Glu318Gly* variant in *PSEN1* had a wide range of 95% confidence intervals due to small sample size. Other variants had much weaker effect sizes, and those variants with hazard ratio under 1.9 were not statistically significant. When adjusted for APOE*4, hazard ratios in the model did not modify the magnitude of HR (Fig. 1).

We subsequently performed a genewise analysis combining all mutations in each gene. This analysis revealed that variants in the *PSEN1* (HR = 6.2; 4.6–8.2) and *GRN* (HR = 9.9; 5.0–19.6) genes significantly increased the risk of dementia (data not shown).

### Comparisons against sequence database

To determine whether these variants were previously identified in independent external population sets, we checked allele frequencies in European Americans and African Americans in the Exome Variant Server (http://evs.gs.washington.edu/EVS/). As of January 2013, seven of the 12 variants were present and four were novel mutations. Of the remaining, five (*p.Gly206Ala* variant in *PSEN1*) were absent in the Exome Variant Server database but was previously reported by us, and four were novel (Table S1).

## Discussion

We resequenced exons from five candidate genes associated with familial dementias (*APP*, *PSEN1*, *PSEN2*, *GRN*, and *MAPT*). Resequencing of these target genes was conducted in 183 Caribbean Hispanic patients for a study of familial dementia from families with four or more affected individuals, originating in the Dominican Republic and Puerto Rico. The raw sequence data were analyzed for potentially pathogenic variants. Potentially pathogenic variants were then genotyped in other sampled family members to determine whether the variant segregated within the family. When the variant was present in probands and relatives, we then genotyped the variant in an independent group of cognitively normal controls from the same Hispanic populations to determine population allelic frequencies.

Most familial dementias, including AD, are multifactorial disorders with multiple putative genetic influences. While the late-onset form of the disease is more common, the genetics of early-onset AD, defined by an age of onset before 65 years have been well studied although gaps remain (van Broeckhoven 1995). Variants in *PSEN1*, *PSEN2*, and *APP* account for nearly 50% of cases (van Broeckhoven et al. 1992; Cruts and van Broeckhoven 1998a,b1998b; Rogaeva et al. 2001). Mutated versions of these genes increase the ratio of A*β*42:A*β*40 ratios through either increased A*β* production or improper clearance, thereby contributing to the characteristic neuritic plaques that represent the neuropathology hallmarks of the disease (Rademakers et al. 2003a). Although these variants tend to have strong signals, direct exome resequencing of multiplex families with dementia have the power to detect rare variants with somewhat weaker signals that might otherwise be missed. It also enables the establishment of segregation patterns within families as compared to the sequencing of unrelated individuals. Applying these approaches to Caribbean Hispanic populations is valuable because they have an increased frequency of dementia compared with similarly aged whites of European ancestry, and the number of putative variants tends to be relatively small.

A previous family study involving these five genes identified mutations among Caucasians and Hispanics (Cruchaga et al. 2012) including the *p.Gly206Ala* variant in *PSEN1*. However, only a few Hispanic families were included. We discovered that 11 individuals from seven families were sequenced or genotyped in the study by Cruchaga et al. as well as the present study, and 11 mutations were identified. For nine individuals, the identified mutations were identical in both experiments. For two individuals, Cruchaga et al. (2012) reported that one individual had a *p.Asp135Val* variant in *GRN* (pathogenicity unknown) and the other had two mutations (*p.Pro85Ala* variant in *GRN* and *p.Arg62His* variant in *PSEN2*) that were likely to be nonpathogenic. The reason for the differences between the two experiments stems from the filtering algorithm where tolerant variants were not further genotyped. Among patients from South America a similar study confirmed the presence of additional mutations (Jin et al. 2012). A number of mutations have been reported at low frequencies (Cruts et al. 2012), necessitating further family studies of early onset Alzheimer disease with a large number of cases. Furthermore, confirmed mutations for early-onset AD are highly penetrant (Sherrington et al. 1995; Tanzi and Bertram 2001; St George-Hyslop and Petit 2005).

Several studies have examined *PSEN1* mutations in other Hispanic populations other than from the Dominican Republic (Morelli et al. 1998; Ramirez-Duenas et al. 1998; Arango et al. 2001; Bertoli Avella et al. 2002). Only one founder mutation (p.Leu174Met variant in exon 6) was reported among Cubans, stemming from an ancestral Iberian founder (Bertoli Avella et al. 2002). This mutation was described as autosomal dominant and completely penetrant in 40 relatives. Other previously reported Hispanic mutations had been discovered in individuals from the Netherlands, Belgium, and Italy yet only one of the mutations (p.Ala260Val in exon 8) was shown in vivo to increase A*β*42 levels.

The *p.Gly206Ala* variant in *PSEN1* was found to be the most frequent variant in this population. Characteristic of the *p.Gly206Ala* variant in *PSEN1* is the variability in age at onset, even within the same family. We previously described this mutation and its related functional effects on A*β* production. While the *p.Glu318Gly* variant in *PSEN1* has also been previously described, it was thought to be nonpathogenic due to lack of an effect on *PSEN1 N*-terminus–*C*-terminus proximity or on *PSEN1*–*APP* interactions as reported for other pathogenic mutations (Berezovska et al. 2005). However, this does not exclude the possibility that the *p.Glu318Gly* variant in *PSEN1* could represent a genetic risk factor as it may be in LD with variants in the promotor region (Helisalmi et al. 2000) or elsewhere, or it is a risk factor in a very small subset of familial form of AD. Based on the three carrier families, the present study support allelic association, and the hazard ratio was elevated but with a wide confidence interval. Thus, these rare, novel variants with incomplete penetrance pose a challenge for clinical interpretation.

*GRN* and *MAPT* mutations are typically associated with FTLD, yet some individuals with mutated forms of these genes have exhibited phenotypes that are indistinguishable from AD (Rademakers et al. 2003b; Doran et al. 2007; Gijselinck et al. 2008; Fenoglio et al. 2009; Jin et al. 2012) as was observed in this study. There are no confirmed reports of *GRN* or *MAPT* mutations in pathologically confirmed AD. On the contrary, several articles reported these two genes' variants associated with clinical diagnoses of AD. This phenotype continuum spectrum involving dementias as FTLD and AD may complicate the diagnostic algorithm; thus, especially in families with more than one form of dementia, assessments of genetic mutations could improve differential diagnosis.

## Supporting Information

**Table S1.** Allele frequencies in the Exome Variant Server.
