# Supplementary material for: Disease-related mutations among Caribbean Hispanics with familial dementia
Source: Mol Genet Genomic Med. 2014 Jun 4;2(5):430–7. doi: 10.1002/mgg3.85 (PMC4190878; doi:10.1002/mgg3.85)
Supplement: Supplementary file 1 — Table S1. Allele frequencies in the Exome Variant Server. [file mgg30002-0430-SD1.docx]

**Online Supplement Table 1. Allele frequencies (%) in the Exome Variant Server**

|  | | |  |
| --- | --- | --- | --- |
|  | **European** | **African** | |
|  | **American (%)** | **Americans (%)** | |
| *PSEN1* |  |  | |
| *p.Gly206Ala* | NA* | NA | |
| *p.Glu318Gly* | 1.872 | 0.431 | |
| *PSEN2* |  |  | |
| *p.Ile235Phe* | NA | NA | |
| *p.Pro301Ala* | 0.07 | 11.235 | |
| *p.Ala344Val* | NA | NA | |
| *GRN* |  |  | |
| *p.Cys222Tyr* | NA | NA | |
| *p.Val519Met* | 0 | 0.023 | |
| *MAPT* |  |  | |
| *p.Ser318Leu* | 0.151 | 17.227 | |
| *p.Ile468Thr* | 0 | 0.726 | |
| *APP* |  |  | |
| *p.Ser614Gly* | 0 | 1.748 | |
| *p.Ala344Val*** | 0.012 | 0 | |
| *p.Val340Met* | NA | NA | |
| ** NA - Variants not observed in the EVS | | | |
| * EVS variant observed was *Ala344Thr* | | | |
